# Jens Kossmann 1963-2023 – a scientist with a passion for plant biology and people

**DOI:** 10.3389/fpls.2023.1266078

**Published:** 2023-08-23

**Authors:** James R. Lloyd, Uwe Sonnewald

**Affiliations:** ^1^ Institute for Plant Biotechnology, Department of Genetics, Stellenbosch University, Stellenbosch, South Africa; ^2^ Division of Biochemistry, Department of Biology, Friedrich-Alexander-University Erlangen-Nürnberg, Erlangen, Germany

**Keywords:** Jens Kossmann, obituary, starch, carbon metabolism, plants

Professor Jens Kossmann was the Specialty Chief Editor of the Plant Biotechnology section at Frontiers in Plant Science from its inception until his death in March 2023 at the age of 59. He ran the section with great enthusiasm and was an important driver of its success. This obituary is designed to celebrate his contribution to plant science as well as his role as a mentor and friend.

Jens’ career spanned three countries and many different projects. He studied Agricultural Science as an undergraduate at the University of Göttingen, and later received a master degree in microbiology where he identified his first gene (Kossmann et al., 1989) which led to a lifelong passion for molecular biological research. His first positions working with plants were as doctoral student and then principal investigator, starting at the Institute für Genbiologische Forschung in Berlin and then continuing at the Max Planck Institute for Molecular Plant Physiology in Potsdam. In 2001 he became the director of the Plant Science department at the Risø National Laboratory in Denmark; but this mainly administrative role did not suit his passion for driving research in plant science. Because of this, in 2004 he moved to South Africa to become director at the Institute for Plant Biotechnology at Stellenbosch University where he spent the rest of his career. Just after he started there the National Research Foundation in South Africa established a program of funding research chairs to try and improve the nation’s science base. He was awarded one of the first of these in the country, which allowed him to concentrate on his major interest, understanding plant carbohydrate metabolism.

In his early career he embraced the possibilities that were becoming available due to the emergence of molecular biology. The establishment of plant transformation in the 1980’s had led to the ability to manipulate gene expression in transgenic plants through the use of overexpression and antisense constructs. As plant genome sequences were unavailable at that time, Jens spent much effort identifying DNA that encoded the protein he wished to manipulate. From today’s perspective, it is very easy to underestimate how much work and brainpower was required to isolate a single gene and Jens was a true master in developing new and sophisticated screening methods. He benefited greatly from what he learned in his studies, especially during his master’s project, as he knew that for every question there was a microorganism that offered the answer; it just had to be found. We remember many night-long discussions where the distillation of diverse thoughts about gene identification strategies led to the birth of unconventional ideas for screening which he could never wait to implement. He always looked back at this time with a great fondness and it was probably one of the happiest phases of his career, where he both established himself as a scientist and made many friends with whom he would collaborate for the remainder of his life.

His major work involved studying the synthesis and degradation of starch, which is the main carbon store in most plants. In leaves it acts as a short term carbon store and is synthesized during the day and degraded at night, while in storage organs, such as seeds and tubers, it accumulates in much higher amounts and is used for long term storage. Studying starch metabolism is important as it is used as an industrial feedstock, influences plant growth and is the major source of calories in the human diet.

In Jens’ early career he was an important international figure involved in isolating genes encoding starch biosynthetic enzymes. He used some of these, encoding isoforms of starch synthase and starch branching enzyme, to demonstrate that they affect starch structure and industrial properties (Kossmann et al., 1991; Abel et al., 1996; Flipse et al., 1996; Kossmann et al., 1999; Lloyd et al., 1999). These discoveries enabled him, and others, to establish a startup biotechnology company which tried to exploit this knowledge by producing plants with altered starch properties for improved industrial use. This company employed many former students from the institutes where he worked and eventually became part of Bayer Crop Science.

His most important contribution to science was probably the identification of the glucan, water dikinase enzyme that introduces phosphate groups into starch in many diverse plant species, and which is also involved in starch degradation in both photosynthetic tissue and potato tubers (Lorberth et al., 1998; Yu et al., 2001; Ritte et al., 2002; Nashilevitz et al., 2009; Mdodana et al., 2019). The inhibition of starch degradation in potato tubers is significant as it affects a process known as cold-induced sweetening where tubers stored at low temperatures degrade starch and accumulate reducing sugars. When potato tubers are fried these react with amino acids to form small amounts of the neurotoxin acrylamide (Mottram et al., 2002; Stadler et al., 2002). The discovery of the glucan, water dikinase led to one of the first biotechnological strategies to repress this process. This gene has also been incredibly important for research into starch metabolism more generally and has been used by many research groups to help better understand the processes of starch synthesis, starch degradation and starch granule formation.

The observation that repression of glucan, water dikinase affects starch degradation led Jens to initiate projects examining this process in more detail in potato as the pathway was poorly understood at the time. He was involved in identifying some of the first enzymes demonstrated to be important in leaf starch degradation *in vivo*, such as a β-amylase (Scheidig et al., 2002) and a disproportionating enzyme (Lloyd et al., 2004). The work in potato was overtaken by other groups using Arabidopsis who have since produced a detailed model of starch degradation in leaves of that plant (Smith and Zeeman, 2020). He used this to identify targets in potato to examine if the pathway is similar between these two species. One example of this was the examination of STARCH EXCESS 4 glucan phosphatase proteins, which remove starch phosphate and which are also important for leaf starch degradation in Arabidopsis (Kötting et al., 2009). Interestingly repression of these enzymes not only affects leaf starch degradation in potatoes, but also increases the phosphate content in potato tuber starch, which alters its potential industrial uses (Samodien et al., 2018).

Identifying processes that influence the rate of starch synthesis could be used to increase the caloric content of some crops. Jens was involved in many projects designed to identify steps that could influence this, for example the identification of genes encoding proteins which lead to the formation of ADP-glucose - the substrate used to form the starch polymer (Müller-Röber et al., 1990; Tauberger et al., 2000). Another way to increase starch contents would be to increase the supply of carbon to the plastid where starch is synthesized. An approach to achieve this involved expression of a yeast invertase in either the apoplast or cytosol of potato plants (Sonnewald et al., 1997). This did not increase starch contents, but did alter tuber size and number. His work also studied other genes that indirectly affect starch metabolism. A plastidial inorganic pyrophosphatase is essential for starch to be synthesized, but also affects many other plastidial processes such as abscisic acid synthesis. Repression of this protein decreases starch accumulation and makes the plants more sensitive to dehydration as they are unable to regulate their stomata (George et al., 2010). He was involved in at least two projects that successfully increased starch production, both of which involved manipulation of nucleotide pools. The first of these repressed the plastidial form of the enzyme adenylate kinase (Regierer et al., 2002) and led to increases in starch amounts in potato tubers. The second reduced activity of UMP-synthase (Geigenberger et al., 2005) and did not increase starch concentration in tubers, but did increase tuber production which would lead to increased starch yield.

The institute in South Africa where he spent most of his career had been established prior to his arrival as a link between academia and the local sugar industry. This led to many applied projects aimed at improving sugarcane. These included the development of plants with reduced amounts of starch (Ferreira et al., 2008) as the small starch content of sugarcane stems can form gels during sucrose isolation. He was also involved in projects leading to the development of improved genetic transformation technologies (van der Vyver et al., 2013; van Beek et al., 2018) and the selection of abiotic stress resistant plants (Masoabi et al., 2018). Increasing sucrose contents in sugarcane is, of course, a major aim of industry and he was involved also in several projects aimed at examining this (Roussouw et al., 2010; van der Merwe et al., 2010; Gabriel et al., 2021).

Although his best known work examined carbon metabolism, he was always interested in developing new projects. When he arrived in South Africa he started using various omics approaches to examine the ways that plant growth promoting substances function (Gouws et al., 2012; Pholo et al., 2018). These studies have provided some idea as to why the substance lumichrome improves growth and has identified potential genetic targets for manipulation. He was also involved in studies trying to improve the nutritional content of plants through influencing vitamin c contents by genetic engineering (Keller et al., 1999; Cronje et al., 2011) or by examining nutrients in microgreens grown under different light conditions (Xonti et al., 2020).

The breadth of his research demonstrated Jens’ fascination with biology. However, he did not just want to understand how genes affected biological processes, but was also driven to try and apply that knowledge to engineer crops with improved yield that can be used to improve human health and alleviate inequality. The most recent Specialty Grand Challenge article (Lloyd and Kossmann, 2021) that he helped write for the Plant Biotechnology section sums his views up well, and he saw the section as a way to try and support researchers developing applied projects. He was also a fantastic collaborator and was involved in many international projects working with other researchers around the world. Within the work environment he was especially interested in helping to develop students and academic staff members so that they could fulfill their potential. He allowed members of his group to follow their own ideas, always encouraging them with helpful suggestion and linking them to collaborators who could aid the projects they had initiated. We will miss him and remember him as a friend who loved discussing his many interests with us, whether sport (he always loved football, but after his move to South Africa developed a surprising passion for cricket also), travel, music, food, politics or the people he had met throughout his career. His death is a great loss to the community, but many of his former students now sit in senior positions in both industry and academia and are themselves guiding the next generation. We like to think because of this that his legacy will continue into the future.

## Author contributions

JL: Writing – original draft. US: Writing – review & editing.
